# Hypothesis testing, type I and type II errors

**DOI:** 10.4103/0972-6748.62274

**Published:** 2009

**Authors:** Amitav Banerjee, U. B. Chitnis, S. L. Jadhav, J. S. Bhawalkar, S. Chaudhury

**Affiliations:** Department of Community Medicine, D. Y. Patil Medical College, Pune, India; 1 Department of Psychiatry, RINPAS, Kanke, Ranchi, India

**Keywords:** Effect size, Hypothesis testing, Type I error, Type II error

## Abstract

Hypothesis testing is an important activity of empirical research and evidence-based medicine. A well worked up hypothesis is half the answer to the research question. For this, both knowledge of the subject derived from extensive review of the literature and working knowledge of basic statistical concepts are desirable. The present paper discusses the methods of working up a good hypothesis and statistical concepts of hypothesis testing.

Karl Popper is probably the most influential philosopher of science in the 20^th^century (Wulff *et al*., 1986). Many scientists, even those who do not usually read books on philosophy, are acquainted with the basic principles of his views on science. The popularity of Popper’s philosophy is due partly to the fact that it has been well explained in simple terms by, among others, the Nobel Prize winner Peter Medawar (Medawar, 1969). Popper makes the very important point that empirical scientists (those who stress on observations only as the starting point of research) put the cart in front of the horse when they claim that science proceeds from observation to theory, since there is no such thing as a pure observation which does not depend on theory. Popper states, “… the belief that we can start with pure observation alone, without anything in the nature of a theory, is absurd: As may be illustrated by the story of the man who dedicated his life to natural science, wrote down everything he could observe, and bequeathed his ‘priceless’ collection of observations to the Royal Society to be used as inductive (empirical) evidence.

## STARTING POINT OF RESEARCH: HYPOTHESIS OR OBSERVATION?

The first step in the scientific process is not observation but the generation of a hypothesis which may then be tested critically by observations and experiments. Popper also makes the important claim that the goal of the scientist’s efforts is not the verification but the falsification of the initial hypothesis. It is logically impossible to verify the truth of a general law by repeated observations, but, at least in principle, it is possible to falsify such a law by a single observation. Repeated observations of white swans did not prove that all swans are white, but the observation of a single black swan sufficed to falsify that general statement (Popper, 1976).

## CHARACTERISTICS OF A GOOD HYPOTHESIS

A good hypothesis must be based on a good research question. It should be simple, specific and stated in advance (Hulley *et al*., 2001).

### Hypothesis should be simple

A simple hypothesis contains one predictor and one outcome variable, e.g. positive family history of schizophrenia increases the risk of developing the condition in first-degree relatives. Here the single predictor variable is positive family history of schizophrenia and the outcome variable is schizophrenia. A complex hypothesis contains more than one predictor variable or more than one outcome variable, e.g., a positive family history and stressful life events are associated with an increased incidence of Alzheimer’s disease. Here there are 2 predictor variables, i.e., positive family history and stressful life events, while one outcome variable, i.e., Alzheimer’s disease. Complex hypothesis like this cannot be easily tested with a single statistical test and should always be separated into 2 or more simple hypotheses.

### Hypothesis should be specific

A specific hypothesis leaves no ambiguity about the subjects and variables, or about how the test of statistical significance will be applied. It uses concise operational definitions that summarize the nature and source of the subjects and the approach to measuring variables (History of medication with tranquilizers, as measured by review of medical store records and physicians’ prescriptions in the past year, is more common in patients who attempted suicides than in controls hospitalized for other conditions). This is a long-winded sentence, but it explicitly states the nature of predictor and outcome variables, how they will be measured and the research hypothesis. Often these details may be included in the study proposal and may not be stated in the research hypothesis. However, they should be clear in the mind of the investigator while conceptualizing the study.

### Hypothesis should be stated in advance

The hypothesis must be stated in writing during the proposal state. This will help to keep the research effort focused on the primary objective and create a stronger basis for interpreting the study’s results as compared to a hypothesis that emerges as a result of inspecting the data. The habit of post hoc hypothesis testing (common among researchers) is nothing but using third-degree methods on the data (data dredging), to yield at least something significant. This leads to overrating the occasional chance associations in the study.

## TYPES OF HYPOTHESES

For the purpose of testing statistical significance, hypotheses are classified by the way they describe the expected difference between the study groups.

### Null and alternative hypotheses

The null hypothesis states that there is no association between the predictor and outcome variables in the population (There is no difference between tranquilizer habits of patients with attempted suicides and those of age- and sex- matched “control” patients hospitalized for other diagnoses). The null hypothesis is the formal basis for testing statistical significance. By starting with the proposition that there is no association, statistical tests can estimate the probability that an observed association could be due to chance.

The proposition that there is an association — that patients with attempted suicides will report different tranquilizer habits from those of the controls — is called the alternative hypothesis. The alternative hypothesis cannot be tested directly; it is accepted by exclusion if the test of statistical significance rejects the null hypothesis.

### One- and two-tailed alternative hypotheses

A one-tailed (or one-sided) hypothesis specifies the direction of the association between the predictor and outcome variables. The prediction that patients of attempted suicides will have a higher rate of use of tranquilizers than control patients is a one-tailed hypothesis. A two-tailed hypothesis states only that an association exists; it does not specify the direction. The prediction that patients with attempted suicides will have a different rate of tranquilizer use — either higher or lower than control patients — is a two-tailed hypothesis. (The word tails refers to the tail ends of the statistical distribution such as the familiar bell-shaped normal curve that is used to test a hypothesis. One tail represents a positive effect or association; the other, a negative effect.) A one-tailed hypothesis has the statistical advantage of permitting a smaller sample size as compared to that permissible by a two-tailed hypothesis. Unfortunately, one-tailed hypotheses are not always appropriate; in fact, some investigators believe that they should never be used. However, they are appropriate when only one direction for the association is important or biologically meaningful. An example is the one-sided hypothesis that a drug has a greater frequency of side effects than a placebo; the possibility that the drug has fewer side effects than the placebo is not worth testing. Whatever strategy is used, it should be stated in advance; otherwise, it would lack statistical rigor. Data dredging after it has been collected and post hoc deciding to change over to one-tailed hypothesis testing to reduce the sample size and P value are indicative of lack of scientific integrity.

## STATISTICAL PRINCIPLES OF HYPOTHESIS TESTING

A hypothesis (for example, Tamiflu [oseltamivir], drug of choice in H1N1 influenza, is associated with an increased incidence of acute psychotic manifestations) is either true or false in the real world. Because the investigator cannot study all people who are at risk, he must test the hypothesis in a sample of that target population. No matter how many data a researcher collects, he can never absolutely prove (or disprove) his hypothesis. There will always be a need to draw inferences about phenomena in the population from events observed in the sample (Hulley *et al*., 2001). In some ways, the investigator’s problem is similar to that faced by a judge judging a defendant [Table 1]. The absolute truth whether the defendant committed the crime cannot be determined. Instead, the judge begins by presuming innocence — the defendant did not commit the crime. The judge must decide whether there is sufficient evidence to reject the presumed innocence of the defendant; the standard is known as beyond a reasonable doubt. A judge can err, however, by convicting a defendant who is innocent, or by failing to convict one who is actually guilty. In similar fashion, the investigator starts by presuming the null hypothesis, or no association between the predictor and outcome variables in the population. Based on the data collected in his sample, the investigator uses statistical tests to determine whether there is sufficient evidence to reject the null hypothesis in favor of the alternative hypothesis that there is an association in the population. The standard for these tests is shown as the level of statistical significance.

**Table 1:** The analogy between judge’s decisions and statistical tests

Judge’s decision	Statistical test
Innocence: The defendant did not commit crime	Null hypothesis: No association between Tamiflu and psychotic manifestations
Guilt: The defendant did commit the crime	Alternative hypothesis: There is association between Tamiflu and psychosis
Standard for rejecting innocence: Beyond a reasonable doubt	Standard for rejecting null hypothesis: Level of statistical significance (à)
Correct judgment: Convict a criminal	Correct inference: Conclude that there is an association when one does exist in the population
Correct judgment: Acquit an innocent person	Correct inference: Conclude that there is no association between Tamiflu and psychosis when one does not exist
Incorrect judgment: Convict an innocent person.	Incorrect inference (Type I error): Conclude that there is an association when there actually is none
Incorrect judgment: Acquit a criminal	Incorrect inference (Type II error): Conclude that there is no association when there actually is one

## TYPE I (ALSO KNOWN AS ‘α’) AND TYPE II (ALSO KNOWN AS ‘β’)ERRORS

Just like a judge’s conclusion, an investigator’s conclusion may be wrong. Sometimes, by chance alone, a sample is not representative of the population. Thus the results in the sample do not reflect reality in the population, and the random error leads to an erroneous inference. A type I error (false-positive) occurs if an investigator rejects a null hypothesis that is actually true in the population; a type II error (false-negative) occurs if the investigator fails to reject a null hypothesis that is actually false in the population. Although type I and type II errors can never be avoided entirely, the investigator can reduce their likelihood by increasing the sample size (the larger the sample, the lesser is the likelihood that it will differ substantially from the population).

False-positive and false-negative results can also occur because of bias (observer, instrument, recall, etc.). (Errors due to bias, however, are not referred to as type I and type II errors.) Such errors are troublesome, since they may be difficult to detect and cannot usually be quantified.

## EFFECT SIZE

The likelihood that a study will be able to detect an association between a predictor variable and an outcome variable depends, of course, on the actual magnitude of that association in the target population. If it is large (such as 90% increase in the incidence of psychosis in people who are on Tamiflu), it will be easy to detect in the sample. Conversely, if the size of the association is small (such as 2% increase in psychosis), it will be difficult to detect in the sample. Unfortunately, the investigator often does not know the actual magnitude of the association — one of the purposes of the study is to estimate it. Instead, the investigator must choose the size of the association that he would like to be able to detect in the sample. This quantity is known as the effect size. Selecting an appropriate effect size is the most difficult aspect of sample size planning. Sometimes, the investigator can use data from other studies or pilot tests to make an informed guess about a reasonable effect size. When there are no data with which to estimate it, he can choose the smallest effect size that would be clinically meaningful, for example, a 10% increase in the incidence of psychosis. Of course, from the public health point of view, even a 1% increase in psychosis incidence would be important. Thus the choice of the effect size is always somewhat arbitrary, and considerations of feasibility are often paramount. When the number of available subjects is limited, the investigator may have to work backward to determine whether the effect size that his study will be able to detect with that number of subjects is reasonable.

## α,β,AND POWER

After a study is completed, the investigator uses statistical tests to try to reject the null hypothesis in favor of its alternative (much in the same way that a prosecuting attorney tries to convince a judge to reject innocence in favor of guilt). Depending on whether the null hypothesis is true or false in the target population, and assuming that the study is free of bias, 4 situations are possible, as shown in Table 2 below. In 2 of these, the findings in the sample and reality in the population are concordant, and the investigator’s inference will be correct. In the other 2 situations, either a type I (α) or a type II (β) error has been made, and the inference will be incorrect.

**Table 2:** Truth in the population versus the results in the study sample: The four possibilities

Truth in the population	Association + nt	No association
Reject null hypothesis	Correct	Type I error
Fail to reject null hypothesis	Type II error	Correct

The investigator establishes the maximum chance of making type I and type II errors in advance of the study. The probability of committing a type I error (rejecting the null hypothesis when it is actually true) is called α (alpha) the other name for this is the level of statistical significance.

If a study of Tamiflu and psychosis is designed with α = 0.05, for example, then the investigator has set 5% as the maximum chance of incorrectly rejecting the null hypothesis (and erroneously inferring that use of Tamiflu and psychosis incidence are associated in the population). This is the level of reasonable doubt that the investigator is willing to accept when he uses statistical tests to analyze the data after the study is completed.

The probability of making a type II error (failing to reject the null hypothesis when it is actually false) is called β (beta). The quantity (1 - β) is called power, the probability of observing an effect in the sample (if one), of a specified effect size or greater exists in the population.

If β is set at 0.10, then the investigator has decided that he is willing to accept a 10% chance of missing an association of a given effect size between Tamiflu and psychosis. This represents a power of 0.90, i.e., a 90% chance of finding an association of that size. For example, suppose that there really would be a 30% increase in psychosis incidence if the entire population took Tamiflu. Then 90 times out of 100, the investigator would observe an effect of that size or larger in his study. This does not mean, however, that the investigator will be absolutely unable to detect a smaller effect; just that he will have less than 90% likelihood of doing so.

Ideally alpha and beta errors would be set at zero, eliminating the possibility of false-positive and false-negative results. In practice they are made as small as possible. Reducing them, however, usually requires increasing the sample size. Sample size planning aims at choosing a sufficient number of subjects to keep alpha and beta at acceptably low levels without making the study unnecessarily expensive or difficult.

Many studies s*et al*pha at 0.05 and beta at 0.20 (a power of 0.80). These are somewhat arbitrary values, and others are sometimes used; the conventional range for alpha is between 0.01 and 0.10; and for beta, between 0.05 and 0.20. In general the investigator should choose a low value of alpha when the research question makes it particularly important to avoid a type I (false-positive) error, and he should choose a low value of beta when it is especially important to avoid a type II error.

## *P* VALUE

The null hypothesis acts like a punching bag: It is assumed to be true in order to shadowbox it into false with a statistical test. When the data are analyzed, such tests determine the *P* value, the probability of obtaining the study results by chance if the null hypothesis is true. The null hypothesis is rejected in favor of the alternative hypothesis if the P value is less than alpha, the predetermined level of statistical significance (Daniel, 2000). “Nonsignificant” results — those with P value greater than alpha — do not imply that there is no association in the population; they only mean that the association observed in the sample is small compared with what could have occurred by chance alone. For example, an investigator might find that men with family history of mental illness were twice as likely to develop schizophrenia as those with no family history, but with a *P* value of 0.09. This means that even if family history and schizophrenia were not associated in the population, there was a 9% chance of finding such an association due to random error in the sample. If the investigator had set the significance level at 0.05, he would have to conclude that the association in the sample was “not statistically significant.” It might be tempting for the investigator to change his mind about the level of statistical significance ex post facto and report the results “showed statistical significance at *P* < 10”. A better choice would be to report that the “results, although suggestive of an association, did not achieve statistical significance (*P* = .09)”. This solution acknowledges that statistical significance is not an “all or none” situation.

## CONCLUSION

Hypothesis testing is the sheet anchor of empirical research and in the rapidly emerging practice of evidence-based medicine. However, empirical research and, ipso facto, hypothesis testing have their limits. The empirical approach to research cannot eliminate uncertainty completely. At the best, it can quantify uncertainty. This uncertainty can be of 2 types: Type I error (falsely rejecting a null hypothesis) and type II error (falsely accepting a null hypothesis). The acceptable magnitudes of type I and type II errors are set in advance and are important for sample size calculations. Another important point to remember is that we cannot ‘prove’ or ‘disprove’ anything by hypothesis testing and statistical tests. We can only knock down or reject the null hypothesis and by default accept the alternative hypothesis. If we fail to reject the null hypothesis, we accept it by default.
